# Structural remodeling impact of osteophytes and Schmorl’s nodes on lumbar vertebral morphometry: anatomical basis for spinal instrumentation

**DOI:** 10.1186/s12891-026-09547-w

**Published:** 2026-01-27

**Authors:** Betül Digilli Ayaş, Rukiye Soyal, Gülay Açar, Aynur Emine Çiçekcibaşı

**Affiliations:** Present Address: Department of Anatomy, Faculty of Medicine, Necmettin Erbakan University, Konya, Türkiye

**Keywords:** Lumbar vertebrae, Morphometry, Pedicle screw fixation, Schmorl’s node, Spinal instrumentation, Vertebral osteophytes

## Abstract

**Background:**

Precise anatomical knowledge is fundamental for successful spinal instrumentation, including pedicle screw fixation and interbody fusion. However, degenerative alterations such as osteophytes and Schmorl’s nodes (SNs) can distort standard bony landmarks. This study aimed to evaluate how these specific degenerative features influence vertebral morphology and to identify anatomical variations relevant to surgical planning and implant selection.

**Methods:**

Ninety dry lumbar vertebrae were analyzed. Linear and angular morphometric parameters were measured using digital calipers and goniometers. Vertebral body areas were calculated using ImageJ. Osteophytes were classified by number, location, shape, and length. The presence of SNs was recorded. Statistical analysis included t-tests, ANOVA, chi-square tests, Pearson correlation, and ROC analysis.

**Results:**

Significant laterality was observed, with right-sided dominance in pedicle length and pars interarticularis height. The presence of SNs was significantly associated with larger vertebral body surface areas (*p* < 0.01) and reduced lamina length (*p* = 0.005), indicating structural remodeling. Notably, osteophyte type and location significantly influenced pedicle angles and chord length (CL), suggesting the need for trajectory considerations during instrumentation planning. ROC analysis revealed that left-sided CL was a moderate predictor for the presence of SNs (AUC = 0.698; cut-off = 44.83 mm).

**Conclusion:**

Osteophytes and SNs are associated with significant morphometric deviations that may have implications for multiple aspects of spinal instrumentation. Specifically, osteophyte patterns were associated with deviations in pedicle angulation, while SN presence correlated with vertebral expansion and posterior element changes, these findings may serve as important morphometric considerations for preoperative planning of instrumentation and interbody procedures. Surgeons should consider these morphometric deviations to optimize implant compatibility and surgical navigation in degenerative spines.

## Introduction

The lumbar spine is the most commonly operated spinal region due to its biomechanical role and high incidence of degenerative disorders [1]. Accurate morphometric data are critical in spinal surgeries such as spinal instrumentation, pedicle screw fixation, laminectomy, and fusion procedures to facilitate preoperative planning and provide a detailed anatomical baseline, which may help in reducing potential complications [2, 3].

Structures like the pedicle, lamina, and pars interarticularis (PI) serve as key anatomical landmarks and are vital for surgical planning [4, 5]. High cortical bone density and biomechanical advantages in the pedicle facilitate screw fixation techniques [6, 7], which require precise knowledge of the pedicle’s width, height, length, and angulation. However, standard morphometric data often represent “normal” anatomy, whereas surgical interventions are frequently performed on spines altered by degenerative processes, where these landmarks may exhibit significant morphometric deviations.

Lamina morphometry provides essential anatomical references for decompressive procedures [8], while PI measurements offer a morphometric baseline to consider during surgical planning to preserve segmental integrity [4, 9, 10]. Additionally, characterizing the vertebral body framework is important for understanding the anatomical space available for implant compatibility in fusion and interbody cage placement [11].

Osteophytes, mostly observed in L4–L5, may encroach upon the anatomical spaces associated with vascular or neural elements, especially anteriorly [12–15]. Schmorl’s nodes (SNs) are degenerative lesions associated with back pain and Scheuermann’s disease. Critically, SNs and subtle osteophyte patterns can be precisely characterized in direct dry bone assessments due to the absence of soft tissue interference, providing a clear view of surface topography that complements radiologic imaging [16–20].

Degenerative spinal stenosis, which predominantly affects the lumbar region, is a multifactorial condition primarily characterized by facet joint hypertrophy and ligamentum flavum thickening. However, narrowing of the spinal canal is also significantly influenced by the development of marginal osteophytes and vertebral remodeling. As proposed in the model by Abbas et al. (2017), SNs may indirectly contribute to this process by triggering a degenerative cascade, including disc height loss and segment instability, which indirectly affects vertebral foramen dimensions [9, 15, 17]. Therefore, correlating these degenerative findings with morphometric changes in the vertebral canal is essential for a comprehensive anatomical assessment of the lumbar spine.

Despite the clinical prevalence of these conditions, there is a paucity of data on how specific features such as osteophyte patterns and Schmorl’s nodes (SNs) remodel the anatomical landmarks required for surgical instrumentation. To address this gap, the present study provides a comprehensive morphometric analysis of dry human lumbar vertebrae by categorizing vertebral osteophytes and evaluating their relationship with SN-associated structural changes. Ultimately, this research aims to establish an anatomical framework that may serve as a baseline for surgical planning and anatomical considerations for implant selection by quantifying the morphometric deviations induced by these degenerative features.

## Materials and methods

### Study design

This study was conducted on 90 dry human lumbar vertebrae from the institutional anatomical collection of the Department of Anatomy, Faculty of Medicine, Necmettin Erbakan University. Due to the disarticulated nature of the institutional collection, specific vertebral levels (L1–L5), age, and sex were not recorded. To maintain data integrity and avoid the inherent risks of misclassification, the specimens were analyzed as a collective lumbar group. This approach not only ensured that the morphometric data remained focused on universal remodeling patterns but also allowed for the presentation of pooled data with higher statistical power, avoiding potentially inaccurate level-specific assignments. Exclusion criteria were strictly defined to differentiate between pathological destruction and degenerative remodeling: specimens exhibiting fractures, severe maceration, malignancies, or congenital anomalies disrupting anatomical integrity were excluded. However, vertebrae displaying degenerative features such as osteophytes and SNs were specifically included to assess their morphometric impact.

### Ethical statement

The study protocol was approved by the Necmettin Erbakan University Faculty of Medicine Ethics Committee (Approval No: 2023/4670). As the study utilized anonymized dry bone specimens from an existing institutional collection, the requirement for informed consent was waived.

### Morphometric measurements

Linear measurements were obtained using a digital caliper (0.01 mm precision), and angular measurements were taken using a digital goniometer (1° precision). To ensure accuracy in surface area calculations, digital photographs of the vertebral bodies were taken with a standardized calibration scale, and areas were calculated using ImageJ software (National Institutes of Health, USA). All bilateral measurements were performed on both the right and left sides. All measurements were performed by a single observer.

#### Pedicle

**Fig. 1:**
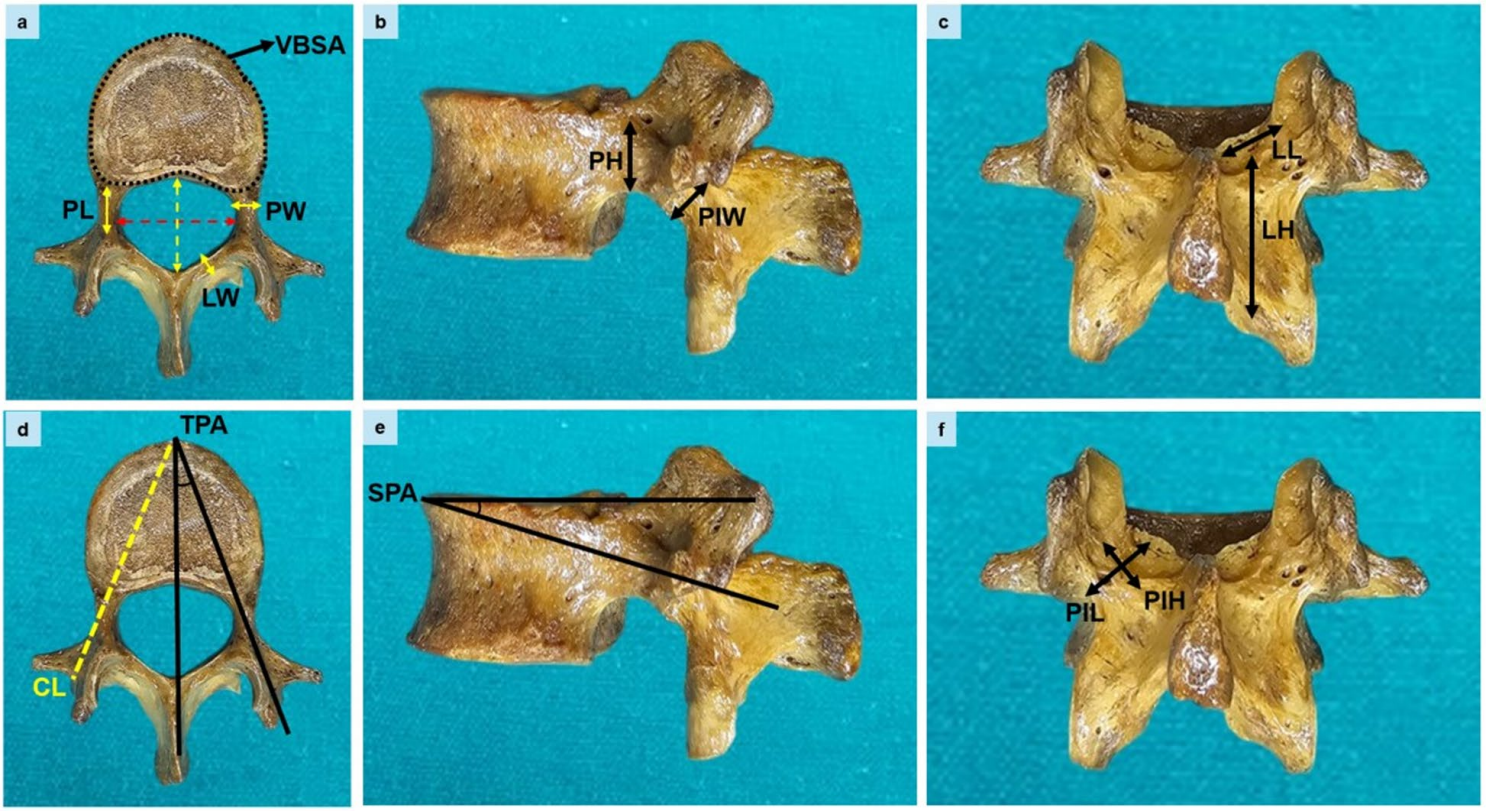
Demonstration of morphometric measurements performed on dry lumbar vertebrae. **a** Superior view. PL: pedicle length, PW: pedicle width, LW: lamina width, yellow bidirectional dashed arrow (VFSD: vertebral foramen sagittal diameter), red bidirectional dashed arrow (VFTD: vertebral foramen transverse diameter), area enclosed by the black dashed line indicates VBSA (vertebral body superior area) **b** Lateral view. PH: pedicle height, PIW: pars interarticularis width **c** Posterior view. LL: lamina length, LH: lamina height **d** Superior view. CL: chord length, TPA: transverse pedicle angle **e** Lateral view. SPA: sagittal pedicle angle **f** Posterior view. PIL: pars interarticularis length, PIH: pars interarticularis height

Width (PW) (Fig. 1a), height (PH) (Fig. 1b), length (PL) (Fig. 1a), transverse pedicle angle (TPA) (Fig. 1d), sagittal pedicle angle (SPA) (Fig. 1e).

#### Posterior elements

Lamina: Width (LW) (Fig. 1a), height (LH) (Fig. 1c), length (LL) (Fig. 1c).

Pars interarticularis: Width (PIW) (Fig. 1b), height (PIH) (Fig. 1f), length (PIL) (Fig. 1f).

#### Surgical trajectory-canal

Chord length (CL, representing screw trajectory) (Fig. 1d), vertebral foramen sagittal and transverse diameters (VFSD, VFTD) (Fig. 1a).

#### Vertebral body

Vertebral body superior and inferior surface areas (VBSA, VBIA) (Fig. 1a).

#### Assessment of degenerative features

Degenerative changes were classified based on established systems to ensure reproducibility:

#### Schmorl’s nodes (SNs)

**Fig. 2:**
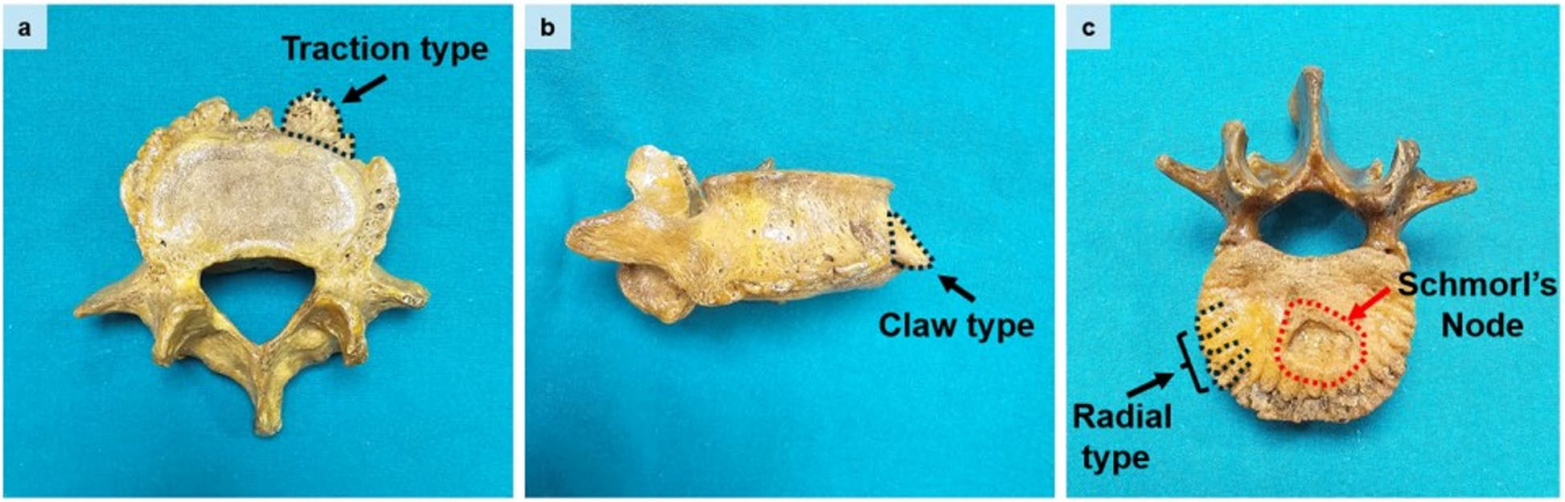
Black arrows indicates osteophytes with different morphological types observed in the lumbar vertebrae. **a** Superior view showing a traction-type osteophyte **b** Lateral view showing a claw-type osteophyte **c** Superior view showing a radial-type osteophyte. The red arrow denotes the presence of a Schmorl’s node

Identified and recorded as present/absent according to Wang et al. [16] (Fig. 2c).

#### Osteophytes

Analyzed based on:

#### Position

Anterosuperior (AS), Anteroinferior (AI), or combined (ASAI) [12].

#### Shape

Classified as traction (Fig. 2a), claw (Fig. 2b), radial type (expanding horizontally) (Fig. 2c) [14].

#### Length

Categorized into five grades based on the following measurements: Type I (< 2 mm), Type II (2–4 mm), Type III (4–6 mm), Type IV (6–8 mm), and Type V (> 8 mm) [21].

### Statistical analysis

Data were analyzed using SPSS v27.0 (IBM Corp., Armonk, NY, USA). Normality distribution was verified using skewness and kurtosis values. For parametric data, paired t-tests were used for side comparisons, and independent t-tests or ANOVA (with Bonferroni post-hoc correction) were used for group comparisons. Categorical variables were analyzed using the Chi-square test. Pearson’s correlation coefficient was used to assess relationships between continuous variables. Intra-observer reliability was confirmed via the intraclass correlation coefficient (ICC) (ICC = 0.980; 95% CI: 0.958–0.990; *p* < 0.001). Finally, Receiver Operating Characteristic (ROC) analysis was performed to evaluate the diagnostic predictive value of CL for the presence of SNs, determining the area under the curve (AUC) and optimal cut-off values. Statistical significance was set at *p* < 0.05.

## Results

### Morphometric laterality and asymmetry

Among the 90 lumbar vertebrae analyzed, laterality significantly influenced multiple morphometric parameters. The right side demonstrated significantly greater values in PL, LH, PIH, CL, TPA, and SPA (all *p* < 0.05). Conversely, PW was significantly greater on the left side (*p* = 0.023). No other bilateral differences reached statistical significance (Table 1).

**Table 1:** Right–left comparison of lumbar morphometric parameters

	Right (*n* = 90) Mean ± SD	Left (*n* = 90) Mean ± SD	t	*p*	Cohen’s d [95% CI]
PW	9.94 ± 3.78	10.19 ± 3.99	-2.31	0.023*	-0.24 [-0.45, -0.03]
PH	13.77 ± 1.84	13.89 ± 1.77	-1.01	0.312	-0.10 [-0.31, 0.10]
PL	5.42 ± 1.01	5.18 ± 1.07	4.75	0.000*	0.50 [0.28, 0.71]
LW	4.23 ± 1.76	4.29 ± 1.84	-0.77	0.438	-0.08 [-0.28, 0.12]
LH	20.24 ± 3.20	19.85 ± 2.84	2.51	0.014*	0.26 [0.05, 0.47]
LL	7.70 ± 1.83	7.69 ± 1.90	0.09	0.921	0.01 [-0.19, 0.21]
PIW	8.79 ± 1.55	8.70 ± 1.51	0.74	0.456	0.07 [-0.12, 0.28]
PIH	6.68 ± 1.93	6.39 ± 1.90	6.26	0.000*	0.66 [0.43, 0.88]
PIL	13.91 ± 2.14	13.88 ± 2.05	0.16	0.871	0.01 [-0.18, 0.22]
CL	46.08 ± 3.64	45.31 ± 3.84	5.02	0.000*	0.52 [0.30, 0.74]
TPA	12.03 ± 1.72	11.82 ± 1.76	4.67	0.000*	0.49 [0.27, 0.71]
SPA	5.81 ± 1.26	5.62 ± 1.23	4.49	0.000*	0.47 [0.25, 0.69]

*CI* Confidence interval, *PW* Pedicle width, *PH* Pedicle height, *PL* Pedicle length, *LW* Lamina width, *LH* Lamina height, *LL* Lamina length, *PIW* Pars interarticularis width, *PIH* Pars interarticularis height, *PIL* Pars interarticularis length, *CL* Chord length, *TPA* Transverse pedicle angle, *SPA* Sagittal pedicle angle, Mean ± SD: Mean ± standard deviation, n: sample size

*Values in boldface indicate statistical significance *p* < 0.05, t: paired samples t statistic, Cohen’s d: effect size

### Impact of schmorl’s nodes on vertebral morphology

**Fig. 3:**
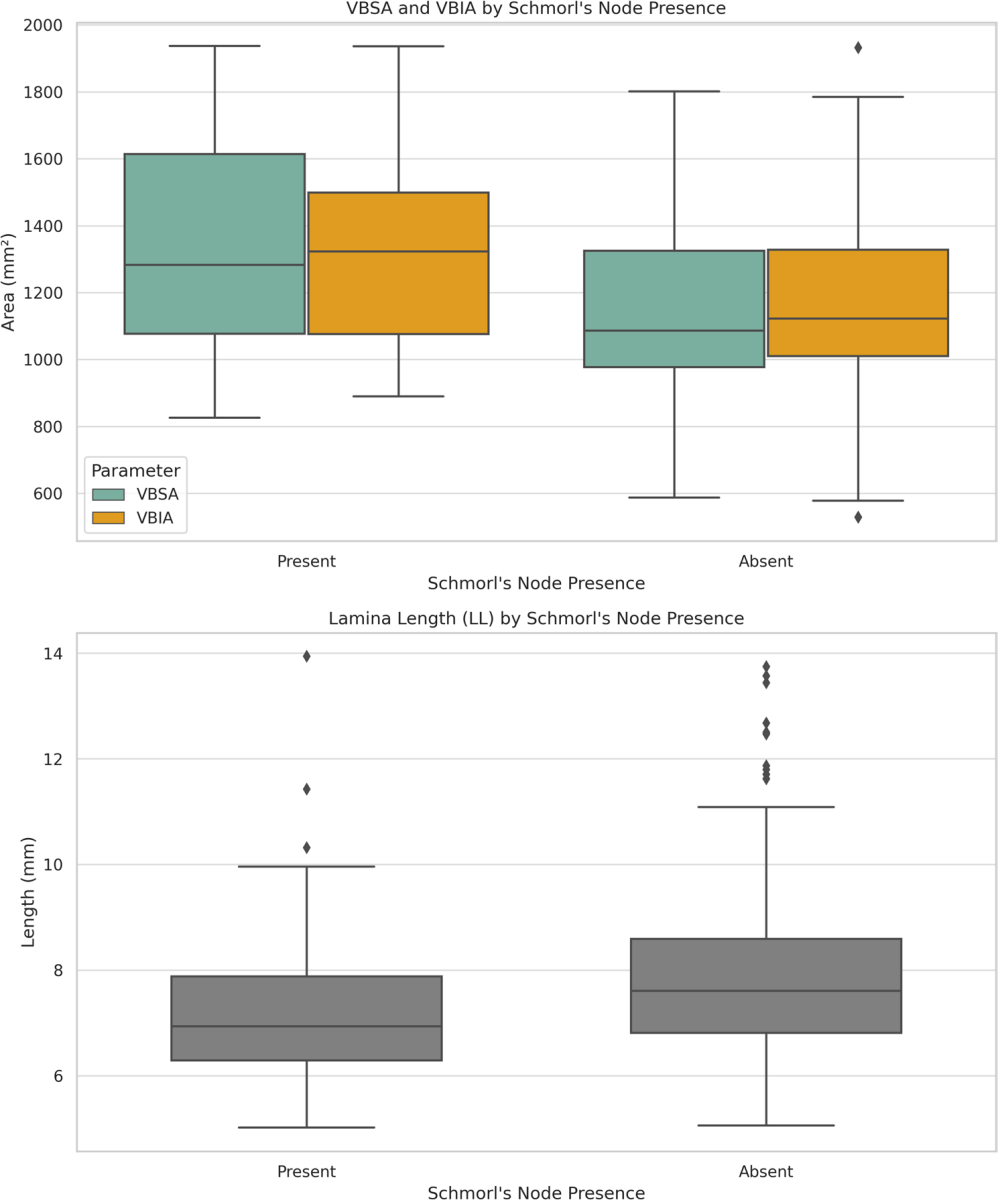
Box plot illustrating the distribution of VBSA (vertebral body superior area), VBIA (vertebral body inferior area), and LL (lamina length) according to the presence or absence of Schmorl’s nodes

SNs were identified in 36.7% of the vertebrae. The presence of SNs was associated with significant structural remodeling; vertebrae with SNs exhibited significantly larger superior (VBSA, *p* < 0.001) and inferior (VBIA, *p* = 0.001) vertebral body surface areas compared to those without. Furthermore, SN presence was associated with a significantly reduced LL (*p* = 0.005) (Fig. 3). Osteophytes were more prevalent in vertebrae with SNs (66.7%) than in those without (54.4%), suggesting a possible degenerative overlap. However, no significant categorical association was found between the presence of SNs and osteophyte characteristics (presence, number, position, type, or length) (Table 2).

**Table 2:** Distribution of vertebral osteophyte characteristics according to the presence of Schmorl’s nodes

		Absent n (%)	Present n (%)	Total n (%)
OE	Absent	26 (45.6%)	11 (33.3%)	37 (41.1%)
	Present	31 (54.4%)	22 (66.7%)	53 (58.9%)
ON	1	6 (19.4%)	6 (27.3%)	12 (22.6%)
	2	9 (29.0%)	8 (36.4%)	17 (32.1%)
	3	7 (22.6%)	2 (9.1%)	9 (17.0%)
	4	4 (12.9%)	2 (9.1%)	6 (11.3%)
	5	2 (6.5%)	1 (4.5%)	3 (5.7%)
	6	1 (3.2%)	2 (9.1%)	3 (5.7%)
	7	2 (6.5%)	1 (4.5%)	3 (5.7%)
OP	AS	9 (29.0%)	4 (18.2%)	13 (24.5%)
	ASAI	15 (48.4%)	12 (54.5%)	27 (50.9%)
	AI	7 (22.6%)	6 (27.3%)	13 (24.5%)
OT	Traction	7 (22.6%)	5 (22.7%)	12 (22.6%)
	Claw	23 (74.2%)	14 (63.6%)	37 (69.8%)
	Radial	1 (3.2%)	3 (13.6%)	4 (7.5%)
OL	Type I	1 (3.2%)	4 (18.2%)	5 (9.4%)
	Type II	11 (35.5%)	5 (22.7%)	16 (30.2%)
	Type III	9 (29.0%)	9 (40.9%)	18 (34.0%)
	Type IV	6 (19.4%)	2 (9.1%)	8 (15.1%)
	Type V	4 (12.9%)	2 (9.1%)	6 (11.3%)

*OE* Osteophyte existence, *ON* Osteophyte number, *OP* Osteophyte position, *OT* Osteophyte shape type, *OL* Osteophyte length, *AS* Anterosuperior, *AI* Anteroinferior, *ASAI* Anterosuperior and anteroinferior, *n *Sample size

### Diagnostic predictive value of chord length

**Fig. 4:**
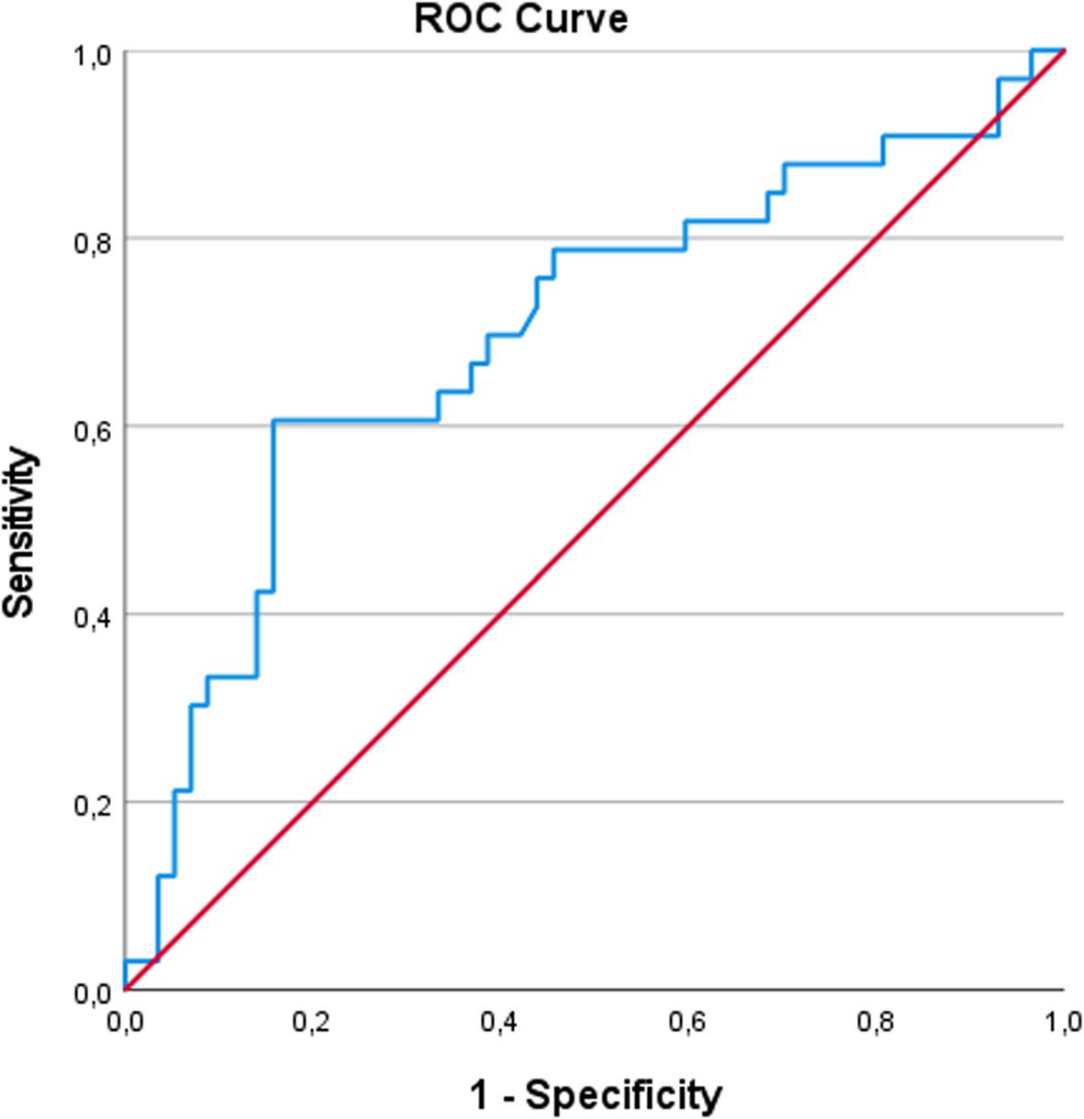
ROC curve analysis of the left-sided CL (chord length) parameter for detecting Schmorl’s nodes. AUC was 0.698 (*p* = 0.002, 95% CI: 0.580–0.816), with a cut-off value of 44.83 mm

ROC analysis evaluated the diagnostic performance of CL in detecting the presence of SNs. Notably, left-sided CL demonstrated moderate diagnostic accuracy (AUC = 0.698, *p* = 0.002, 95% CI: 0.580–0.816). The optimal cut-off value for predicting SN presence was calculated as 44.83 mm. These findings suggest that CL elongation may serve as a surrogate marker for SN-associated vertebral remodeling (Fig. 4).

### Influence of osteophyte characteristics on surgical landmarks

Osteophyte morphology (number, position, shape, and length) showed significant associations with critical surgical dimensions:

#### Osteophyte number

**Fig. 5:**
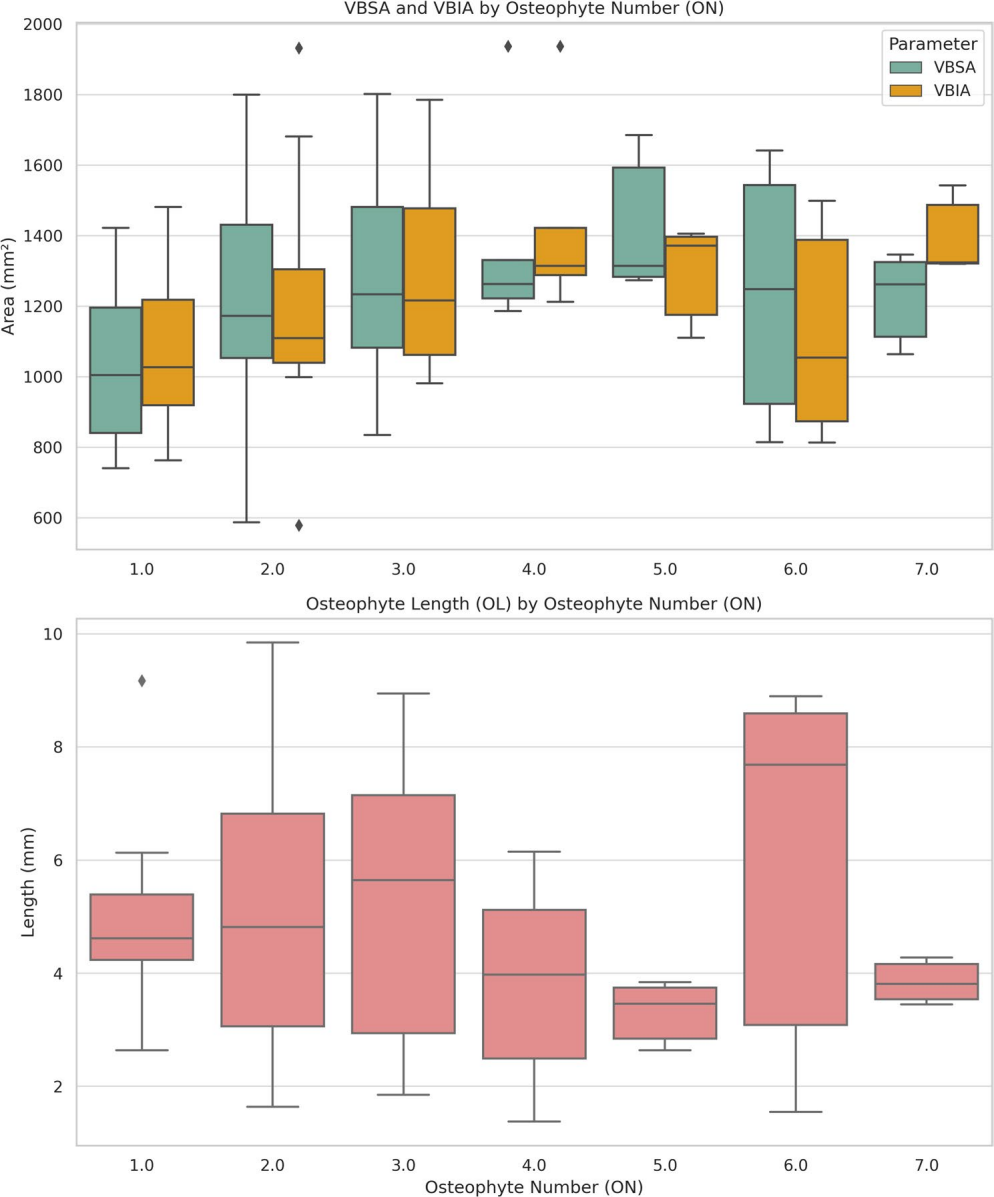
Box plot illustrating the distribution of VBSA (vertebral body superior area), VBIA (vertebral body inferior area), and OL (osteophyte length) according to ON (osteophyte number)

A higher osteophyte burden correlated with vertebral expansion. Vertebrae with four osteophytes had significantly larger VBSA (*p* = 0.025) and VBIA (*p* = 0.012) compared to those with a single osteophyte. Conversely, OL was greater in vertebrae with fewer osteophytes (1–2) compared to those with five (*p* < 0.05) (Fig. 5).

#### Position

**Fig. 6:**
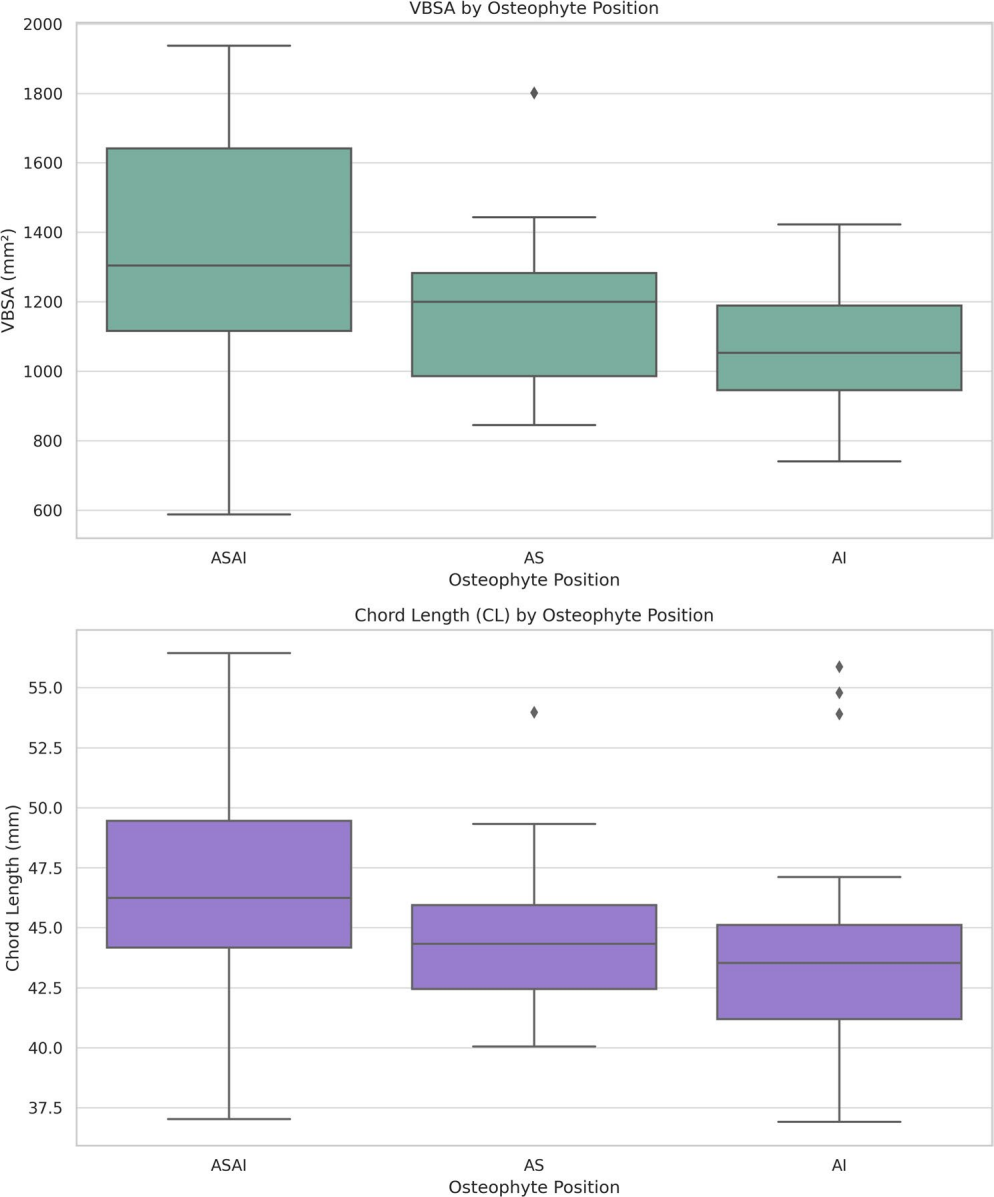
Box plot illustrating the distribution of VBSA (vertebral body superior area) and CL (chord length) according to osteophyte position. ASAI: combined anterosuperior and anteroinferior location, AS: anterosuperior location, AI: anteroinferior location

ASAI osteophytes were associated with significantly greater VBSA (*p* < 0.001) and longer CL (*p* = 0.009) compared to isolated AI osteophytes (Fig. 6).

#### Shape

**Fig. 7:**
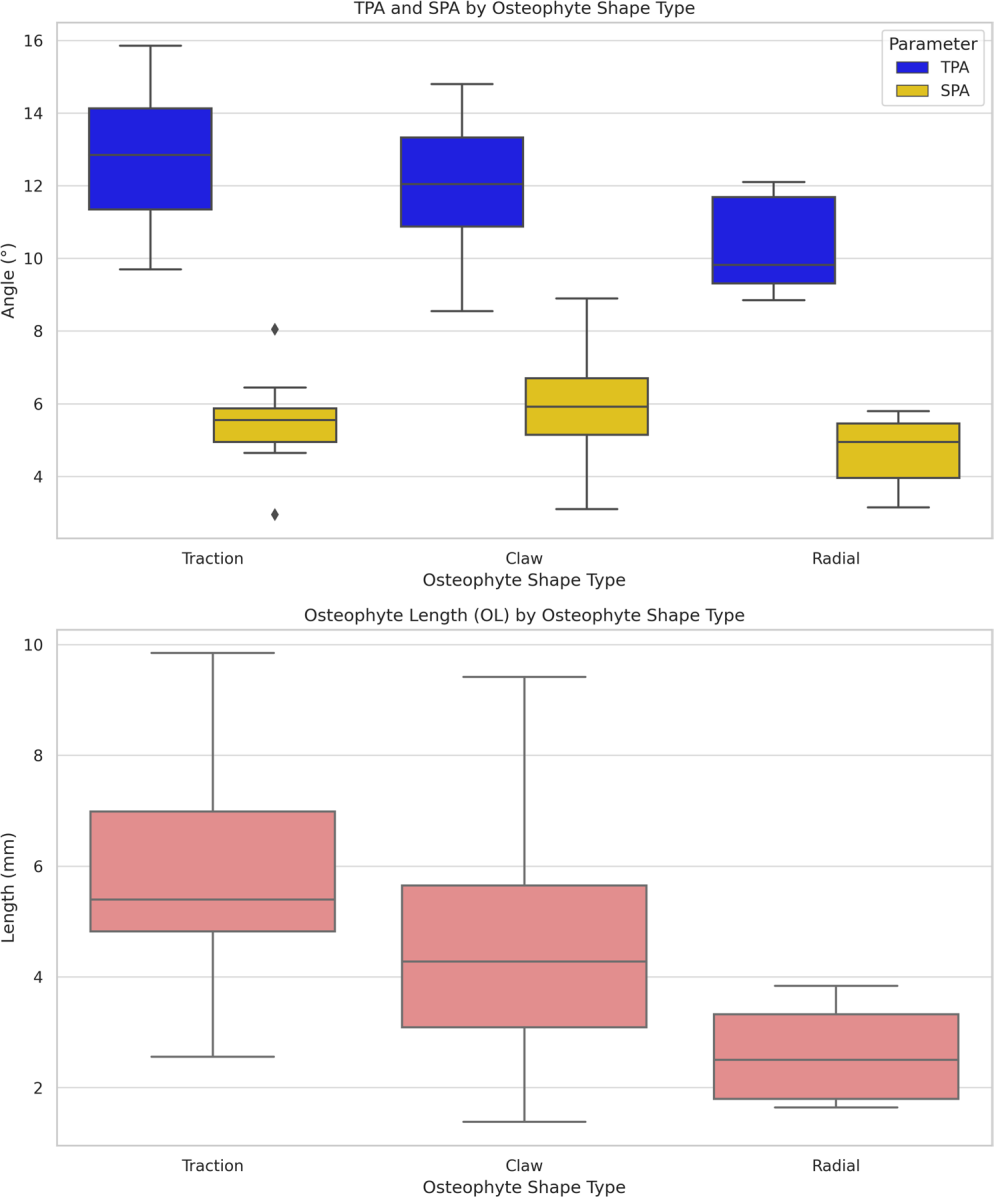
Box plot illustrating the distribution of TPA (transverse pedicle angle), SPA (sagittal pedicle angle), and OL (osteophyte length) according to osteophyte shape type

Osteophyte shape significantly influenced pedicle angulation. Traction and claw-type osteophytes were linked to higher TPA and OL values than radial types (for TPA: *p* = 0.001 and *p* = 0.013; for OL: *p* = 0.001 and *p* = 0.023, respectively). Additionally, claw-type osteophytes showed a higher SPA value than the radial type (*p* = 0.017) (Fig. 7).

#### Length

**Fig. 8:**
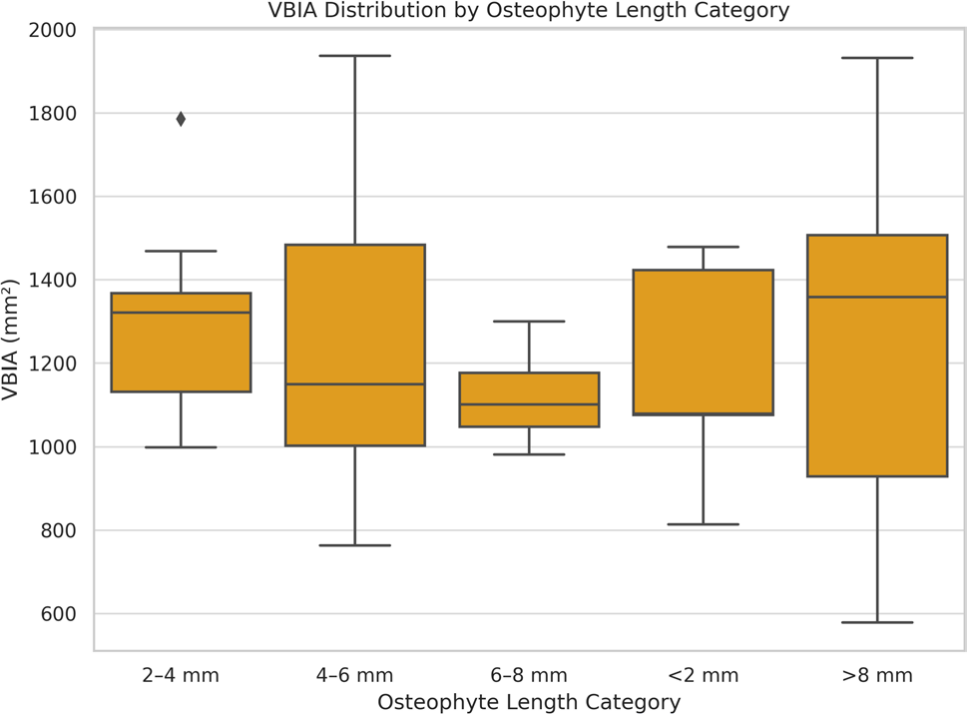
Box plot illustrating the distribution of VBIA (vertebral body inferior area) according to osteophyte length categories

Osteophyte length classification influenced the inferior surface area, with Type II osteophytes associated with larger VBIA compared to Type IV (*p* = 0.004) (Fig. 8).

### Correlations among parameters

Pearson’s correlation analysis revealed strong morphometric interdependencies. CL showed strong positive correlations with PH, LH, PIL, VBSA, VBIA, and VFTD. Regarding pedicle dimensions, PW correlated positively with LL, PIW, PIL, and VFTD, but negatively with LH. TPA showed positive correlations with PL, LW, LL, and VFTD. VFSD showed positive correlations with PL and LL, and a negative correlation with PIL. Full correlation coefficients are detailed in Table 3.

**Table 3:** Pearson correlation matrix among vertebral morphometric parameters

		1	2	3	4	5	6	7	8	9	10	11	12	13	14
1. PW	*r*	1													
	*p*														
2. PH	**r**	− 0.165*	**1**												
	**p**	**0.027**													
3. PL	**r**	− 0.180*	− 0.037	**1**											
	**p**	**0.016**	0.620												
4. LW	**r**	0.130	− 0.054	0.517**	**1**										
	**p**	0.082	0.468	**0.000**											
5. LH	**r**	− 0.502**	0.338**	0.270**	0.109	**1**									
	**p**	**0.000**	**0.000**	**0.000**	0.146										
6. LL	**r**	0.487**	− 0.336**	0.294**	0.412**	− 0.295**	**1**								
	**p**	**0.000**	**0.000**	**0.000**	**0.000**	**0.000**									
7. PIW	**r**	0.355**	0.260**	− 0.097	− 0.002	− 0.002	0.124	**1**							
	**p**	**0.000**	**0.000**	0.197	0.975	0.975	0.097								
8. PIL	**r**	0.323**	0.374**	− 0.147*	− 0.009	0.155*	0.052	0.368**	**1**						
	**p**	**0.000**	**0.000**	**0.049**	0.908	**0.038**	0.490	**0.000**							
9. CL	**r**	0.121	0.326**	0.065	0.131	0.297**	− 0.044	0.110	,323**	**1**					
	**p**	0.104	**0.000**	0.385	0.081	**0.000**	0.554	0.143	,**000**						
10. VFSD	**r**	**-**,190*	− 0.065	0.431**	0.172*	0.004	0.250**	− 0.147*	− 0.193**	0.076	1				
	**p**	**0.011**	0.388	**0.000**	**0.021**	0.956	**0.001**	**0.049**	**0.010**	0.308					
11. VFTD	**r**	0.364**	0.067	0.187*	0.213**	− 0.168*	0.369**	0.275**	0.095	0.248**	0.370**	1			
	**p**	**0.000**	0.372	**0.012**	**0.004**	**0.024**	**0.000**	**0.000**	0.204	**0.001**	**0.000**				
12. TPA	**r**	0.094	0.061	0.373**	0.533**	0.144	0.278**	0.032	0.028	0.162*	0.182*	0.201**	**1**		
	**p**	0.209	0.420	**0.000**	**0.000**	0.054	**0.000**	0.665	0.709	**0.030**	**0.014**	**0.007**			
13. VBSA	**r**	0.113	0.189**	− 0.296**	− 0.254**	0.035	− 0.213**	0.107	0.207**	0.334**	− 0.168*	− 0.038	− 0.090	**1**	
	**p**	0.113	**0.011**	**0.000**	**0.001**	0.642	**0.004**	0.153	**0.005**	**0.000**	**0.024**	0.618	0.230		
14. VBIA	**r**	0.004	0.274**	− 0.262**	− 0.253**	0.128	− 0.236**	0.113	0.217**	0.384**	− 0.076	− 0.047	− 0.092	0.842**	**1**
	**p**	0.957	**0.000**	**0.000**	**0.001**	0.088	**0.001**	0.130	**0.004**	**0.000**	0.309	0.530	0.220	**0.000**	

*PW*: Pedicle width, *PH* Pedicle height, *PL* Pedicle length, *LW* Lamina width, *LH* Lamina height, *LL* Lamina length, *PIW* Pars interarticularis width, *PIH* Pars interarticularis height, *PIL* Pars interarticularis length, *CL* Chord length, *VFSD* Vertebral foramen sagittal diameter, *VFTD* Vertebral foramen transverse diameter, *TPA* Transverse pedicle angle, *VBSA* Vertebral body superior area, *VBIA* Vertebral body inferior area

Pearson’s correlation coefficient (r) was used, Values in boldface indicate statistical significance *p < 0.05 , **p < 0.01

## Discussion

### Pedicle morphometry and anatomical considerations for instrumentation

Pedicle screw fixation is a widely used technique in spinal surgery due to its biomechanical advantages, and requires precise anatomical knowledge to understand the morphometric boundaries that guide neurovascular safety and optimal screw positioning [3]. In this context, PW emerges as one of the most critical parameters in defining the anatomical limits for appropriate screw diameter [22]. Our findings indicate significant laterality, with the left PW being significantly greater than the right. This aligns with dry bone studies by Khair et al. [6] and Patil and Bhuiyan [23], suggesting that in some populations, the left pedicle may present a wider morphometric framework for screw placement. However, other studies by Khan and Zvikomborero [1], Alam et al. [3], and Bonczar et al. [11] reported no significant side differences. Hanarwut et al. [7] noted a higher PW value on the right side only at the L5 level. Additionally, Güleç et al. [22] and Matsukawa et al. [24] demonstrated a gradual increase in PW from L1 to L5 using CT imaging.

Although pedicle PH was found to be higher on the left side in our study, this difference was not statistically significant. Our findings are consistent with those of Khair et al. [6] and similar results were reported in studies by Khan and Zvikomborero [1] and Bonczar et al. [11] where no significant side differences were observed. In contrast, Alam et al. [3] and Patil and Bhuiyan [23] reported higher PH values on the right side, while Hanarwut et al. [7] found higher PH on the right at L1 and on the left at L5. Additionally, Güleç et al. [22] and Matsukawa et al. [24] noted a decreasing trend in PH from L1 to L5.

In all studies, PH was found to be greater than PW, indicating that pedicle width represents the more constrained morphometric dimension when considering the anatomical space available for screw diameter selection. PL was significantly higher on the right side (*p* < 0.001). Due to the limited data on PL in the lumbar region, this finding provides essential anatomical data regarding the morphometric boundaries that may serve as a reference for screw length considerations.

TPA and SPA represent key morphometric indicators for understanding pedicle orientation and the anatomical boundaries related to neurovascular safety [22, 23]. In our study, both angles were significantly greater on the right (*p* < 0.001), consistent with Patil and Bhuiyan [23]. Alam et al. [3] found left-dominant TPA and right-dominant SPA, while Hanarwut et al. [7] reported right-sided SPA only. The tendency for TPA to increase from L1 to L5, as shown in prior studies [7, 22, 24], highlights the inherent angulation shifts that may serve as an anatomical baseline for preoperative considerations in screw placement.

Accurate assessment of the safe bone corridor is essential to respect the anterior anatomical boundaries of the vertebra. In degenerative cases where standard landmarks are often obscured, CL measurement from the accessory process provides a reliable anatomical guide for defining the safe bone corridor [5, 22, 23]. We found significantly longer right-sided CL (*p* < 0.001), consistent with Patil and Bhuiyan [23]. In contrast, Hanarwut et al. [7] reported longer left-sided CL, while others found no side difference [1, 5]. This asymmetry is morphometrically significant; assuming bilateral symmetry during preoperative planning could potentially result in unintended anterior cortex breaching on the shorter side or suboptimal utilization of the available bone corridor on the longer side. Therefore, the identified variations in CL suggest that morphometric assessment of these landmarks should be side-specific rather than based on averaged anatomical norms.

Although morphometric data on the lamina and PI are limited, their anatomical role as surgical landmarks is well established [4, 7, 8]. In our study, LH and PIH were significantly higher on the right, while LW, LL, PIW, and PIL showed no side difference. Ashish et al. [4] found higher LH and PIW on the left and LL on the right. The lack of significant difference in LW and PIL suggests a morphometric consistency across sides, providing a uniform anatomical reference for preoperative considerations. The asymmetry in PIH and PIW highlights the importance of individual assessment, as these dimensions define the available bony corridor for screw angulation and the morphometric framework supporting the posterior elements.

According to Wolff’s Law, bone undergoes structural remodeling to optimize its load-bearing capacity in response to the specific mechanical stress and vectors it encounters [25]. The observed morphometric laterality in our study-where PW and PH were larger on the left, while PL, TPA, SPA, CL, LH, and PIH were larger on the right-may reflect a systematic structural adaptation to asymmetrical loading patterns. The literature supports our findings by stating that even in non-scoliotic controls, a certain degree of vertebral body and pedicle asymmetry exists [26]. We propose that these morphometric shifts are not random anomalies, but rather systematic structural remodeling responses to degenerative instability. Specifically, the larger PW and PH on the left side could potentially serve as a ‘stabilizing pillar’ to balance the axial loads, whereas the increased PL and angulation (TPA/SPA) on the right side might represent a geometric reorientation to accommodate altered torsional forces.

### Schmorl’s nodes and vertebral remodeling

SN prevalence varies widely (9–75%) depending on imaging modality and sample type [16]. Radiographic rates are lowest (3.8–8.1%), while cadaveric studies may exceed 70%. MRI- and CT-based studies report 9–49.9% and 19% prevalence, respectively [19, 20]. Wang et al. [16] reported a 22% prevalence, particularly in the upper lumbar region, with rates increasing with age. In our study, SNs were observed in 36.7% of vertebrae, aligning with prior data.

Unlike previous studies limited to vertebral depth and width, our analysis presents novel data on superior and inferior surface areas. Our study is among the first to comprehensively link SNs with global vertebral morphometry rather than just endplate pathology. We found that vertebrae with SNs exhibit significantly larger surface areas, which may represent a compensatory biomechanical response to maintain load-bearing capacity despite the loss of disc integrity. For interbody fusion procedures, these findings suggest that the presence of SNs is associated with a widened vertebral surface; this could be a relevant morphometric consideration when evaluating the anatomical space available for cage footprints. Additionally, the association between SNs and reduced LL indicates that posterior anatomical corridors may exhibit distinct morphometric patterns, highlighting the importance of preoperative anatomical awareness to respect the integrity of the pars interarticularis.

In our study, mean VFSD and VFTD values (15.54 ± 2.01 mm and 22.79 ± 2.30 mm, respectively) were consistent with previous reports [3, 4] and reflect the canal’s elliptical shape. Prior studies indicate that VFTD reportedly increases caudally [10, 11] and VFSD shows sex- and level-based variation [9]. While the literature primarily focuses on the relationship between these diameters and gender and level, our study investigated the morphometric influence of SNs and osteophytes. Our results demonstrate that canal diameters were not significantly affected by the presence of SNs or specific osteophyte types, suggesting that these bony remodeling patterns do not directly impact the dimensions of the vertebral canal.

### Predictive value of chord length

A novel contribution of this study is the predictive capacity of CL for SN presence. The ROC analysis identified that a left-sided CL > 44.83 mm is a moderate predictor of SNs. Within a morphometric context, an increased chord length trajectory may serve as a potential anatomical indicator of underlying endplate alterations, such as Schmorl’s nodes. These findings suggest the importance of a detailed preoperative anatomical review of endplate integrity and vertebral morphology when such measurements are encountered on preoperative imaging. This provides an additional layer of anatomical awareness for the surgeon during the evaluation of the degenerative lumbar spine.

### The influence of osteophytes on surgical trajectories

Due to variability in sample types and classification methods, osteophyte assessment lacks standardization. While posterior localization is frequently reported [15], Chanapa et al. [14] found the highest prevalence in the anterosuperior region (30.4%), whereas we observed a predominance of combined ASAI patterns (50.9%). Although traction-type osteophytes were most common in their study (78.5%), claw types were more prevalent in our series (69.8%). While Chanapa et al. [14] reported a mean osteophyte length of 3.47 ± 2.21 mm, our mean osteophyte length (4.84 ± 2.23 mm) was notably higher. This difference suggests a more advanced stage of degenerative remodeling in our specimen group, which may explain the significant impact observed on pedicle angulation and chord length. By detailing the classification system (Type I: <2 mm to Type V: >8 mm), we observed that osteophyte length significantly influenced the inferior vertebral surface area (VBIA). Specifically, Type II osteophytes (2–4 mm) were associated with a larger VBIA compared to Type IV (6–8 mm) (*p* = 0.004). This suggests that structural remodeling and expansion of the vertebral base may occur more prominently during the earlier stages of osteophyte development (Type II) rather than in advanced stages where growth is more longitudinal. From an anatomical perspective, this indicates that even relatively short osteophytes are not merely surface alterations but are indicators of broader morphometric shifts in the vertebral body; such changes may represent relevant considerations for understanding the available anatomical space during interbody cage selection.

Beyond their descriptive features, osteophyte number, position, shape, and length demonstrated significant associations with key morphometric parameters. Vertebrae with four osteophytes showed significantly greater VBSA and VBIA, suggesting an association between osteophyte count and vertebral expansion, whereas five-osteophyte cases exhibited shorter spur lengths, potentially reflecting early-stage or reactive changes rather than advanced degeneration. ASAI-positioned osteophytes corresponded with larger VBSA and longer CL, emphasizing the relevance of osteophyte localization in preoperative anatomical assessment. Claw and traction types were associated with greater pedicle angulation, representing a morphometric deviation from standard anatomical norms. This indicates that the pattern of osteophytic degeneration alters the local anatomical fraemwork. Therefore, anatomical awareness of these remodeling patterns is important during preoperative planning to understand how the safe anatomical corridor may deviate from standard landmarks. Furthermore, we identified a “radial” osteophyte type—a horizontally expanding pattern not widely classified in previous literature. Recognizing this morphotype provides additional anatomical context that may be considered during anterior or lateral surgical approaches.

By identifying the associations between SNs and vertebral body expansion, as well as specific osteophyte types and pedicle angulation, our findings suggest that degenerative changes may serve as a catalyst for a systematic morphometric remodeling process. This offers a plausible biomechanical rationale for the asymmetrical boundaries that a surgeon might encounter during spinal instrumentation. Rather than asserting direct causality, these observations highlight the importance of incorporating anatomical awareness of morphometric degenerative remodeling into preoperative planning to potentially enhance surgical navigation in complex cases.

**Fig. 9:**
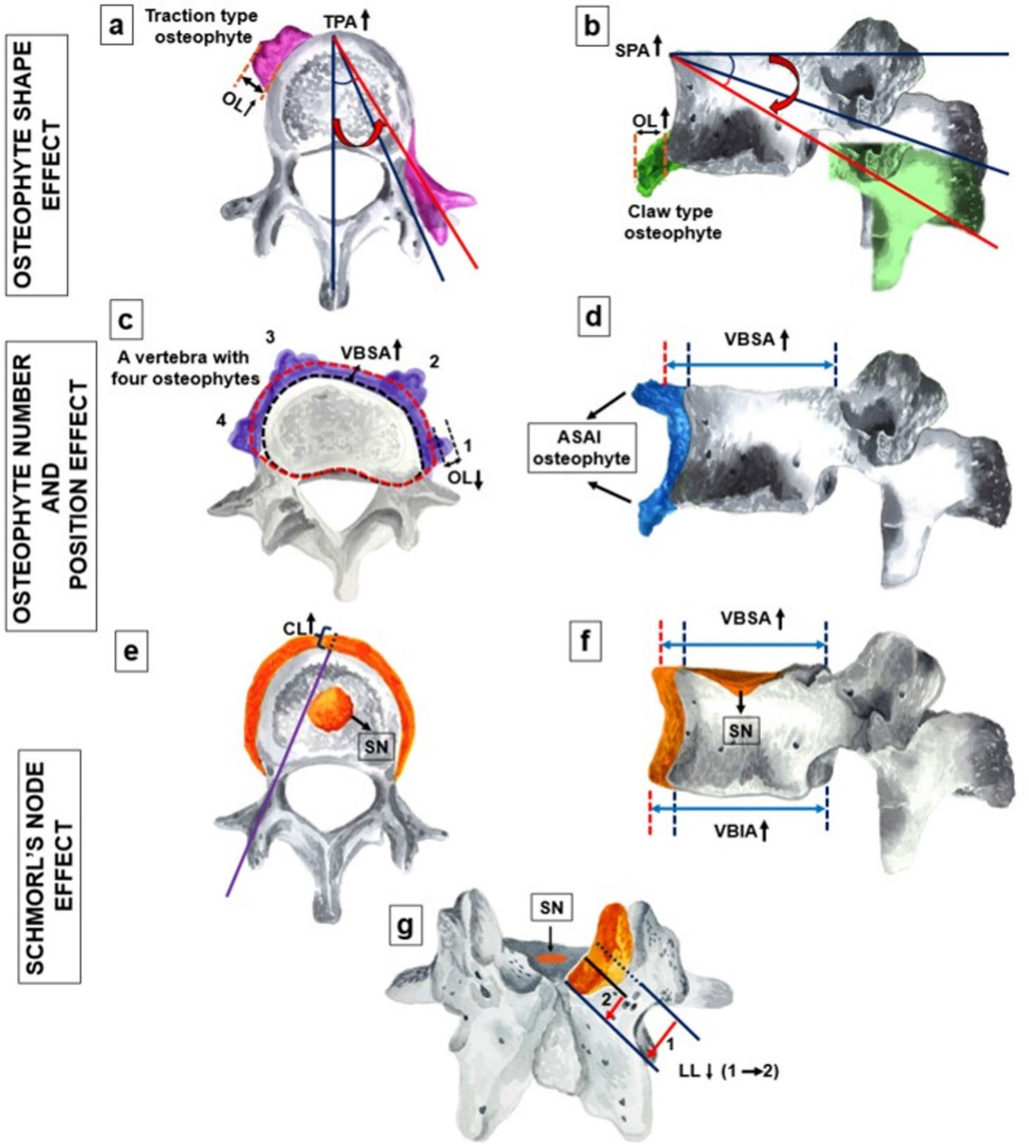
Visual synthesis of degenerative morphometric deviations in the lumbar vertebrae. **a**–**b** Traction and claw-type osteophytes associated with increased TPA, SPA, and OL. **c**–**d** Impact of osteophyte burden and ASAI-positioning on VBSA expansion, noting the reduction in mean OL as osteophyte count increases. **e** ROC-based illustration of increased CL as a morphometric predictor for SN presence. **f**–**g** Significant morphometric shifts in SN-affected vertebrae, including VBSA/VBIA expansion and reduced LL. TPA, transverse pedicle angle; SPA, sagittal pedicle angle; OL, osteophyte length; ASAI, anterior-superior-anterior-inferior; VBSA, vertebral body superior area; VBIA, vertebral body inferior area; CL, chord length; SN, Schmorl’s node; LL, lamina length

**Fig. 10:**
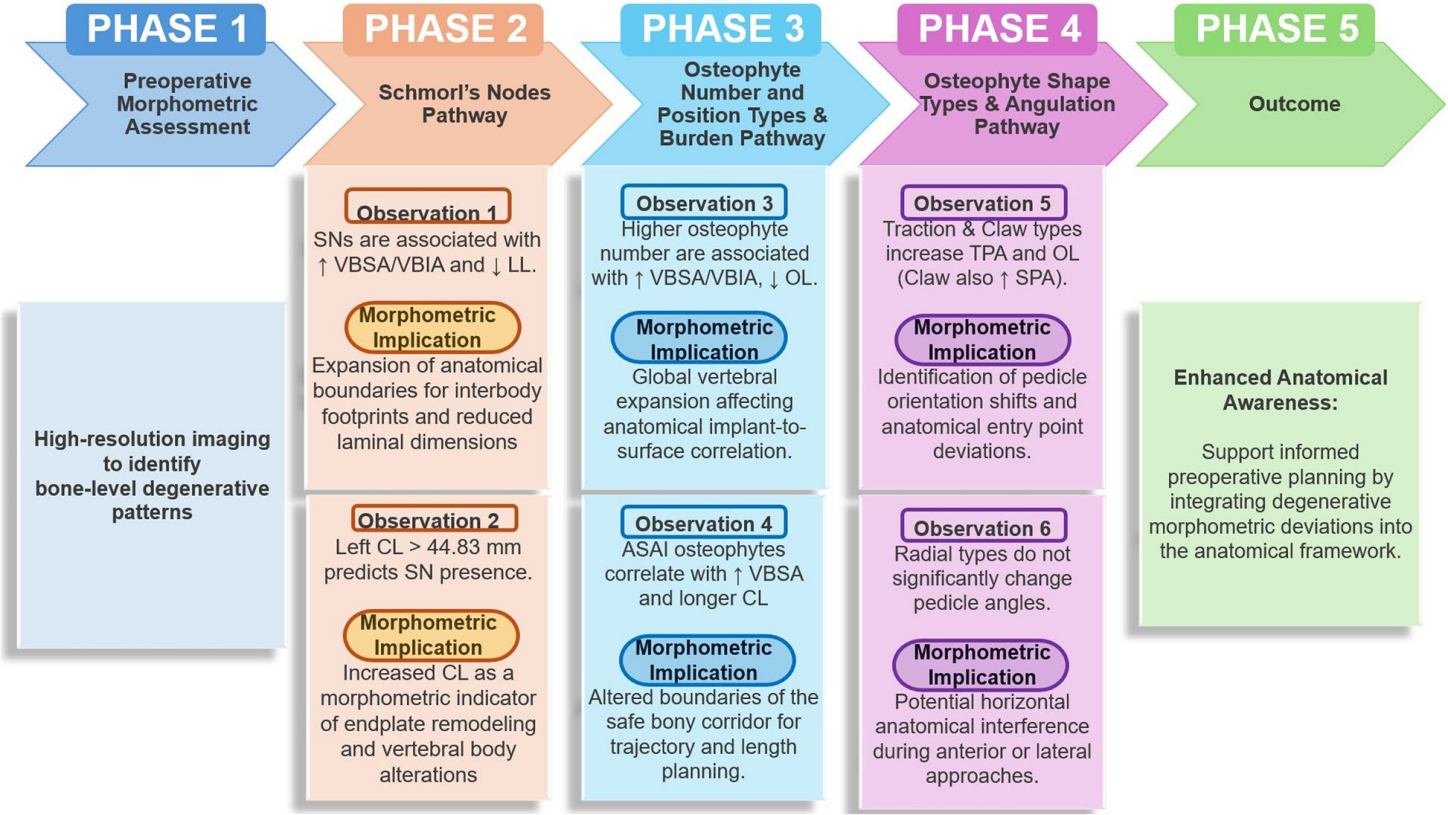
Proposed Anatomical–Morphometric Evaluation Framework for assessing structural deviations due to degenerative changes. Abbreviations: SN: Schmorl’s node; CL: Chord length; VBSA/VBIA: Vertebral body superior/inferior surface area; LL: Lamina length; OL: Osteophyte length; ASAI: Anterosuperior/Anteroinferior osteophytes; TPA: Transverse pedicle angle; SPA: Sagittal pedicle angle

To enhance the clinical utility of our morphometric data, we first provide a visual synthesis of the identified deviations, such as increased pedicle angles and altered chord lengths, in an integrative panel (Fig. 9). Building upon these visual summaries, we propose a standardized Anatomical–Morphometric Evaluation Framework (Fig. 10). This five-phase flow chart provides a systematic framework for translating anatomical remodeling patterns—such as the link between chord length and Schmorl’s node presence—into preoperative anatomical considerations. By doing so, we aim to improve anatomical awareness and provide a morphometric baseline that may support safer navigation during spinal instrumentation in degenerative cases.

### Limitations and strengths

The present study has several limitations. First, the dry bone specimens were obtained from a disarticulated institutional collection, which precluded the identification of specific vertebral levels (L1–L5), age, and sex of the individuals. Consequently, level-specific morphometric trends or sex-based differences could not be established. While these demographic factors influence the severity of degeneration, our study intentionally focused on the geometric end-results of these processes. By quantifying the final morphometric state, we define the actual anatomical boundaries a surgeon encounters intraoperatively, independent of the patient’s demographic background. Furthermore, this methodology allowed for the direct, high-resolution physical measurement of osteophyte types (specifically the ‘radial’ type) and surface areas that are often obscured by soft tissue interference or volume averaging in standard radiological imaging. The biomechanical remodeling responses observed—such as the link between osteophytes and angular changes—likely follow universal adaptive patterns driven by axial loading, thereby retaining their anatomical and clinical validity regardless of the specific vertebral level. Finally, as this was a dry bone study, the lack of associated clinical history or soft tissue elements (ligaments and discs) must be considered when interpreting the results. In particular, since the ligamentum flavum and intervertebral discs play a primary role in the pathophysiology of spinal stenosis, their absence means that our findings are limited to the hard-tissue (bony) component of the spinal corridor. Additionally, without clinical follow-up or patient-reported outcomes, the direct impact of these morphometric deviations on postoperative recovery remains to be validated. Future research should aim to integrate high-resolution three dimensional (3D) computed tomography imaging with clinical data and biomechanical testing to evaluate how these anatomical remodeling patterns may correlate with long-term implant stability and patient outcomes.

## Conclusions

This study highlights that vertebral osteophytes and Schmorl’s nodes are associated with significant morphometric deviations that extend beyond simple surface pathology. The presence of Schmorl’s nodes correlates with increased vertebral body surface area and reduced lamina length, representing important anatomical considerations for interbody cage sizing and posterior decompression planning. Furthermore, specific osteophyte patterns—particularly traction and claw types—are associated with altered pedicle angulation, highlighting the need to account for these morphometric deviations during screw trajectory planning to respect the integrity of the bony corridor. Notably, the capacity of chord length to predict the presence of Schmorl’s nodes offers a novel morphometric indicator for underlying vertebral remodeling. Ultimately, surgeons should integrate these “morphometric considerations” into preoperative planning to support informed anatomical navigation during spinal instrumentation.

## Data Availability

The data sets used and analyzed during the current study are available from the corresponding author on reasonable request.

## Abbreviations

PW: Pedicle width
PH: Pedicle height
PL: Pedicle length
LW: Lamina width
LH: Lamina height
LL: Lamina length
PIW: Pars interarticularis width
PIH: Pars interarticularis height
PIL: Pars interarticularis length
CL: Chord length
VFSD: Vertebral foramen sagittal diameter
VFTD: Vertebral foramen transverse diameter
TPA: Transverse pedicle angle
SPA: Sagittal pedicle angle
VBSA: Vertebral body superior area
VBIA: Vertebral body inferior area
OL: Osteophyte length
SN: Schmorl's node
AS: Anterosuperior
AI: Anteroinferior
ASAI: Anterosuperior and anteroinferior
